# Association of mTOR Polymorphisms with Cancer Risk and Clinical Outcomes: A Meta-Analysis

**DOI:** 10.1371/journal.pone.0097085

**Published:** 2014-05-09

**Authors:** Jianbo Shao, Ying Li, Peiwei Zhao, Xin Yue, Jun Jiang, Xiaohui Liang, Xuelian He

**Affiliations:** 1 Department of CT/MRI, Wuhan Children's Hospital, Hubei, China; 2 Department of Radiology, Wuhan Children's Hospital, Hubei, China; 3 Central laboratory, Wuhan Children's Hospital, Hubei, China; 4 Department of Electrophysiology, Wuhan Children's Hospital, Hubei, China; 5 School of Public Health, Wuhan University, Hubei, China; MD Anderson Cancer Center, United States of America

## Abstract

Genetic polymorphisms in mTOR gene may be associated with cancer risk and clinical outcomes of cancer patients by affecting mTOR gene expression or its activation. However, inconsistent results have been reported. The aim of this study is to systematically evaluate the association between mTOR polymorphisms (rs2295080, rs2536 and rs11121704) and cancer risk as well as clinical outcome by a meta-analysis. We identified 10 eligible studies and extracted data by two investigators. Based on dominant and recessive models, odds ratio (ORs) and 95% confidence intervals (CIs) were calculated by using Stata, version 11 to evaluate the association strength. Our meta-analysis results showed that the wild genotype TT of rs2295080 polymorphism was associated with increased cancer risk under dominant model (OR = 1.24, 95%CI: 1.12–1.36, p<0.0005) in Chinese but not with clinical outcome parameters, while the TT genotype of rs11121704 was associated with poor clinical outcome parameters (OR = 1.53, 95%CI: 1.01–2.32, p = 0.044), such as death, metastasis and resistance to chemotherapy. However, rs2536 may not influence cancer susceptibility. In conclusion, this meta-analysis indicated the common polymorphisms in *mTOR* gene might be genetic risk factors for the carcinogenesis and clinical outcomes of cancer patients. However, further investigation on large population and different ethnicities are warranted.

## Introduction

Mammalian target of rapamycin (mTOR), also known as FRAP (FKBP112-rapamycin-associated protein), was originally discovered about 15 years ago in the study on the mechanism of action of rapamycin [1]. mTOR, a conserved serine/threonine kinase, has been recognized as a central regulator of vital cellular processes through PI3K/AKT/mTOR pathway, such as proliferation, growth, differentiation, survival, and angiogenesis by controlling mRNA translation, ribosome biogenesis, autophagy, and metabolism [2]–[4]. In human, this pathway is frequently activated in many human diseases, including cancers, furthermore, and uncontrolled mTOR signaling had been reported to be associated with poor clinical outcome in lung, cervical, ovarian and esophageal cancers [3], [5]–[11]. In light of the critical role of mTOR in maintaining proper cellular functions, it is biologically plausible that genetic variations in this gene may affect cancer risk and clinical outcome of cancer patients.

mTOR gene is located in chromosome 1q36.2, and there are 3434 genetic polymorphisms within this gene. A few polymorphisms could exert some effects by modulating transcriptional activity, miRNA binding, or splicing [12], e.g. rs2295080 (T>G) in the promoter region, rs2536 in the 3′-untranslated region (3′UTR), and rs17036508 (T>C) in potential splicing site. The polymorphism rs2295080 has been demonstrated to regulate the transcriptional activity and the TT genotypes had higher mTOR mRNA levels [13], and the polymorphism rs2536 was proposed to affect the miRNA binding site activity [12].

Recently, a number of case-control studies reported that the polymorphisms in mTOR gene were associated with individual's susceptibility to cancer risk and clinical outcome [12]–[20], but these studies were limited to modest sample size, different ethnicity, and statistical power. Therefore, we carried out a meta-analysis on all eligible studies to estimate the association between the genetic polymorphisms in mTOR gene and overall cancer risk as well as clinical outcomes. After reviewing literature, we found that besides rs2295080 and rs2536, another polymorphism rs11121704 (T>C) in intron, have been mostly frequently studied, thus, were included in our meta-analysis.

## Materials and Methods

### Literature Research

We searched the electronic database Medline to identify relevant reports by using terms “mTOR”, “polymorphism”, and “cancer” (last search was updated on November 28, 2013). The search was limited to English language articles. Additional studies were identified by reviewing the references of original studies. The studies included in our meta-analysis had to meet the following inclusion criteria: (1) evaluated the association of target mTOR polymorphisms and cancer risk and/or clinical outcomes in patients with cancer; (2) used case-control study or cohort study; (3) provided sufficient information for calculation of odds ratio (ORs) with 95% confidence interval (CI). The following data were extracted from each study: the first author's last name, year of publication, country of origin, type of cancers, number of genotyped cases and controls, number of cases and controls with each genotype, source of control groups (population- or hospital based controls) for cancer risk assessment, and prognosis parameters for clinical outcome assessment. For studies which investigated more than one clinical parameter, such as survival and response to chemotherapy, data were extracted separately for each parameter whenever possible.

### Statistical analysis

For control group of each study, the genotype frequency was assessed for Hardy–Weinberg equilibrium using the Chi-square test (P>0.05). We evaluated the association between the mTOR polymorphisms and cancer risk by calculating the pooled odds ratios (ORs) with 95% confidence intervals (CIs). We estimated the risks of mTOR polymorphisms on cancer by assuming dominant and recessive effects of the rear allele, respectively. Due to the limited data available, we only calculated the pooled OR under the dominant model.

Potential heterogeneity was checked by the χ^2^-based Q-test, if the P value is greater than 0.05 of the Q-test, which indicates a lack of heterogeneity among studies, the summary OR or HR estimate of each study was calculated by the fixed-effects model [21], otherwise, the random-effects model [22] was employed. The significance of the pooled OR or HR was determined by Z-test and P<0.05 was considered as statistically significant.

Sensitivity analyses were performed by removing one study each time to reflect the influence of individual study on the pooled ORs.

Egger's and Begg-Matzumdar tests were used to assess publication bias [23]–[24]. A P value of <0.05 was considered indicative of a statistically significant publication bias.

If the publication bias tests indicated bias existed, the Duval and Tweedie “trim and fill” method was used to adjust the bias [25].

All statistical analyses were done with Stata, version 11 (Stata Corporation, College Station, TX).

## Results

### Characteristics of studies

**Figure 1:**
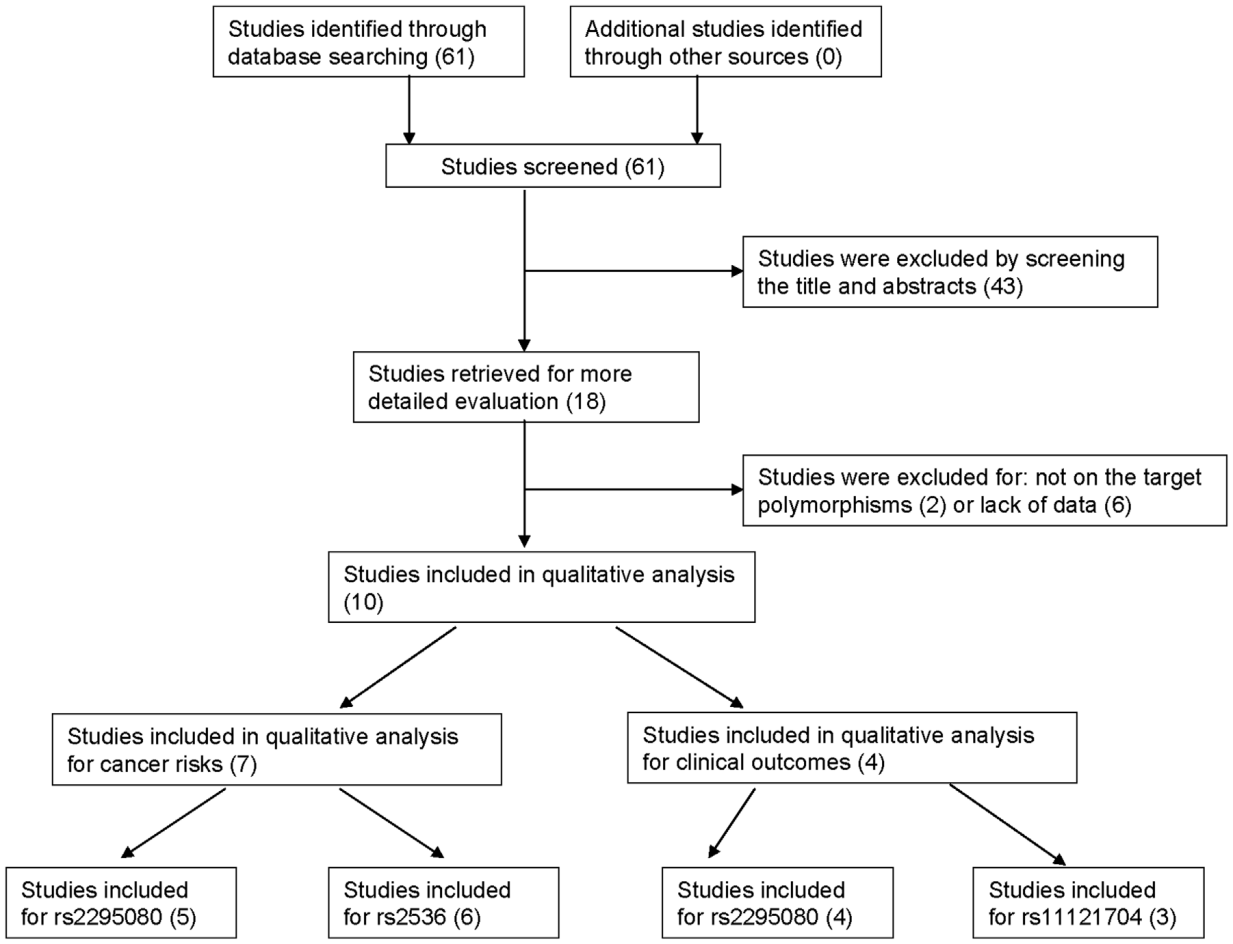
Flow of study identification, inclusion, exclusion.

Through the primary literature research in Pubmed, 61 studies were identified for cancer risk and/or clinical outcome assessment for mTOR polymorphisms. However, after manually screening the titles and abstracts, 43 studies were excluded. The remaining 18 articles were reviewed and, 8 of them were removed due to lack of sufficient data or examing other mTOR polymorphisms but not rs2295080, rs2536 (T>C) and rs11121704 [26]–[33]. Finally, 10 studies were met the inclusion criteria [12]–[20], [35], and 6 studies evaluated the influence on cancer risks [12]–[13], [15]–[18] and 3 assessed the clinical outcomes [19], [20], [35], such as death, metastasis, resistance to chemotherapy, and toxicity, and one examined both [14]. The flow of study identification, inclusion, exclusion was shown in Fig. 1. For cancer risk assessment, all 7 studies were conducted in Chinese population, including 5798 cancer patients and 6244 healthy controls. The types of cancers included renal cell cancer, acute lymphoblastic leukemia, prostate cancer, gastric cancer, and esophageal squamous cell carcinoma. Of the 7 studies, 3 studies used population-based and frequency-matched controls to the cases by the age and region [13], [17]–[18]. All studies used TaqMan SNP Genotyping Assay and randomly repeated assays for genotyping quality control. The genotypes in the controls in all studies were in Hardy-Weinberg equilibrium. For estimating the influence of mTOR polymorphisms (rs2295080 and rs11121704) on clinical outcomes in cancer patients, 4 eligible studies included 1594 cancer patients, were identified: 2 were conducted in USA [19], [20] and two were in China [14], [35]. Two studies in USA evaluating evaluated more than one clinical outcome parameter, and these parameters were separately analyzed as separate observations. All studies extracted DNA from peripheral blood lymphocytes for genotyping except for one study, where tumor tissue was used. The essential information for all studies was shown in Table 1 and 2.

**Table 1:** Study characteristics of the meta-analysis for cancer risk.

Polymorphism	Author	Year	Country	Racial descent	Tumor type	Control source	Case			Control			Genotyping method and quality control
rs2295080							TT	TG	GG	TT	TG	GG	
(T>G)	Cao	2012	China	Chinese	Renal cell cancer	Hospital	454	218	38	438	277	45	Taqman, 5% randomly repeat
	Chen	2012	China	Chinese	Prostate cancer	Hospital	429	209	28	413	259	36	Taqman, 10% randomly repeat
	Huang	2012	China	Chinese	acute lymphocytic leukemia.	Hospital	254	140	23	353	180	21	Taqman, 10% randomly repeat
	Xu	2012	China	Chinese	Gastric Cancer	Hospital	482	246	25	497	305	52	Taqman, duplicated
	Li	2013	China	Chinese	Prostate cancer	Population	653	311	40	617	382	52	Taqman, 5% randomly repeat
rs2536(T>C)							TT	TC	CC	TT	TC	CC	
	Cao	2012	China	Chinese	Renal cell cancer	Hospital	607	99	4	628	128	4	Taqman, 5% randomly repeat
	Chen	2012	China	Chinese	Prostate cancer	Hospital	565	96	5	585	119	4	Taqman, 10% randomly repeat
	Huang	2012	China	Chinese	ALL	Hospital	346	65	6	448	103	3	Taqman, 10% randomly repeat
	Li	2013	China	Chinese	Prostate cancer	Population	804	192	8	894	147	10	Taqman, duplicated repeat
	He	2013	China	Chinese	Gastric Cancer	Population	938	179	8	1019	170	7	Taqman, 5% randomly repeat
	Zhu	2013	China	Chinese	Esophageal Squamous Cell Carcinoma	Population	951	165	7	957	157	7	Taqman, 5% randomly repeat

**Table 2:** Study characteristics of the meta-analysis for clinical outcomes.

Polymorphism	Author	Year	Country	Racial descent	Tumor type	Outcome parameter		Yes	No	
rs2295080							TT	TG+GG	TT	TG+GG
(T>G)	Hildebrandt	2012	USA	Causaian(90%)	Esophageal cancer	Survival	12	71	6	82
	Hildebrandt	2012	USA	Causaian(90%)	Esophageal cancer	Recurrence	14	103	4	50
	Hildebrandt	2012	USA	Causaian(90%)	Esophageal cancer	Response to chemotherapy	10	98	8	54
	Pu	2011	USA	non-Hispanic Caucasian	Lung	Toxicity	7	59	7	91
	Pu	2011	USA	non-Hispanic Caucasian	Lung	Distant progression	7	56	7	94
	Li	2013	China	Chinese	NSCLC	Brain metastasis	58	41	140	78
	Xu	2013	China	Chinese	Gastric Cancer	Distant metastasis	59	39	423	232
rs11121704							TT	TC+CC	TT	TC+CC
(T>C)	Hildebrandt	2012	USA	Causaian(90%)	Esophageal cancer	Survival	9	79	5	87
	Hildebrandt	2012	USA	Causaian(90%)	Esophageal cancer	Recurrence	11	112	3	54
	Hildebrandt	2012	USA	Causaian(90%)	Esophageal cancer	Response to chemotherapy	10	104	4	62
	Pu	2011	USA	non-Hispanic Caucasian	Lung	Toxicity	6	5	5	92
	Pu	2011	USA	non-Hispanic Caucasian	Lung	Distant progression	5	6	6	92
	Li	2013	China	Chinese	NSCLC	Brain metastasis	84	175	175	43

NSCLC, Non-small-cell lung carcinoma.

### Quantitative synthesis

Based on genotyping data available, we noticed that there was a wide variation in the T allele frequency of mTOR rs2295080 polymorphism among cancer patients between Caucasians and Asians (Chinese and Korean),ranging from 0.311 to 0.808. Asians had the higher T allele frequency (0.777–0.808) than Caucasians (0.311–0.353).

**Figure 2:**
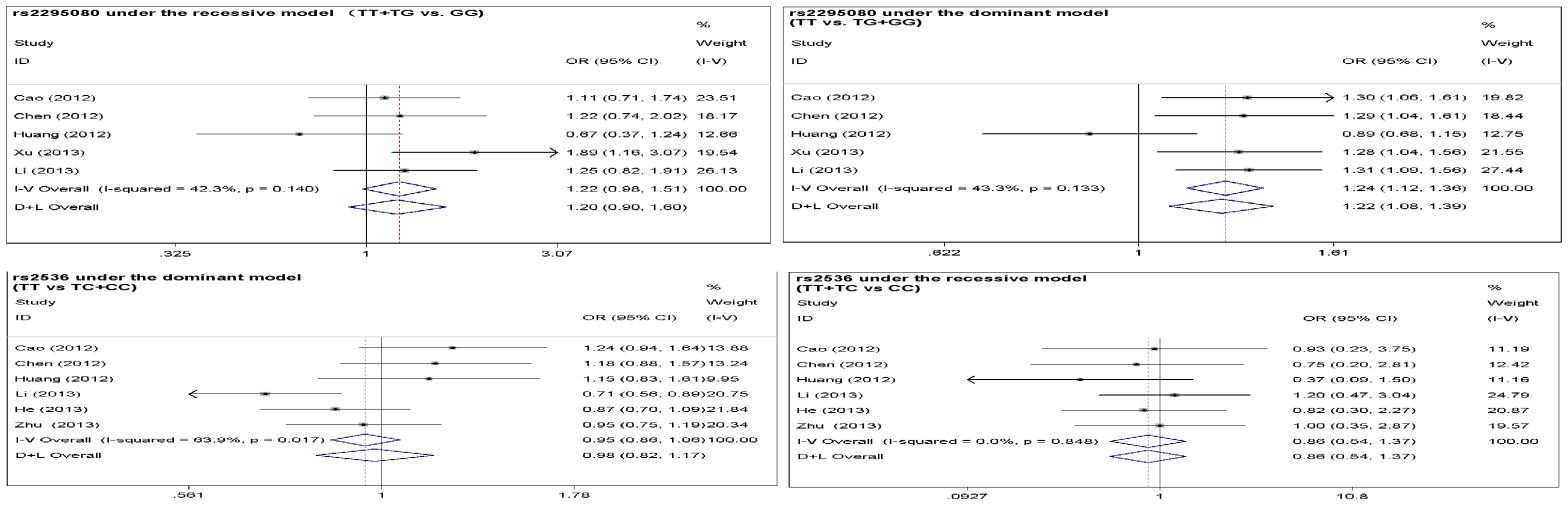
Forest plots of cancer risk with rs2529080 and rs2536 polymorphisms under the dominant and recessive models.

**Figure 3:**
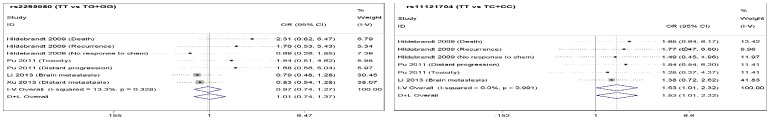
Forest plots of clinical outcomes with the mTOR rs2529080 and rs11121704 polymorphisms under the recessive model.

Overall, our meta-analysis results showed that the wild genotype TT of rs2295080 polymorphism was associated with increased cancer risk under dominant model (OR = 1.24, 95%CI: 1.12–1.36, p<0.0005) (Fig. 2) but not with clinical outcomes (Fig. 3), while the TT genotype of rs11121704 were associated with poor clinical outcome parameters (OR = 1.53, 95%CI: 1.01–2.32, p = 0.044), such as death, metastasis and resistance to chemotherapy (Fig. 3). However, rs2536 was not associated with cancer risk under both dominant and recessive models (Fig. 2).

### Test of heterogeneity and sensitivity

No significant heterogeneity was observed for all analyses except for rs2536 under recessive model (p = 0.017) (Fig. 2). Sensitivity analysis indicated one independent study by Li et al. was the main origin of heterogeneity [35], as the heterogeneity was effectively removed (p = 0.234) while the pooled OR was not significantly changed (95%CI 0.98: 0.82–1.17 vs. 1.04: 0.91–1.20) after deleting this study. In addition, the pooled OR was not qualitatively influenced after removing any single study, indicating our meta-analysis results are stable.

### Publication bias

**Figure 4:**
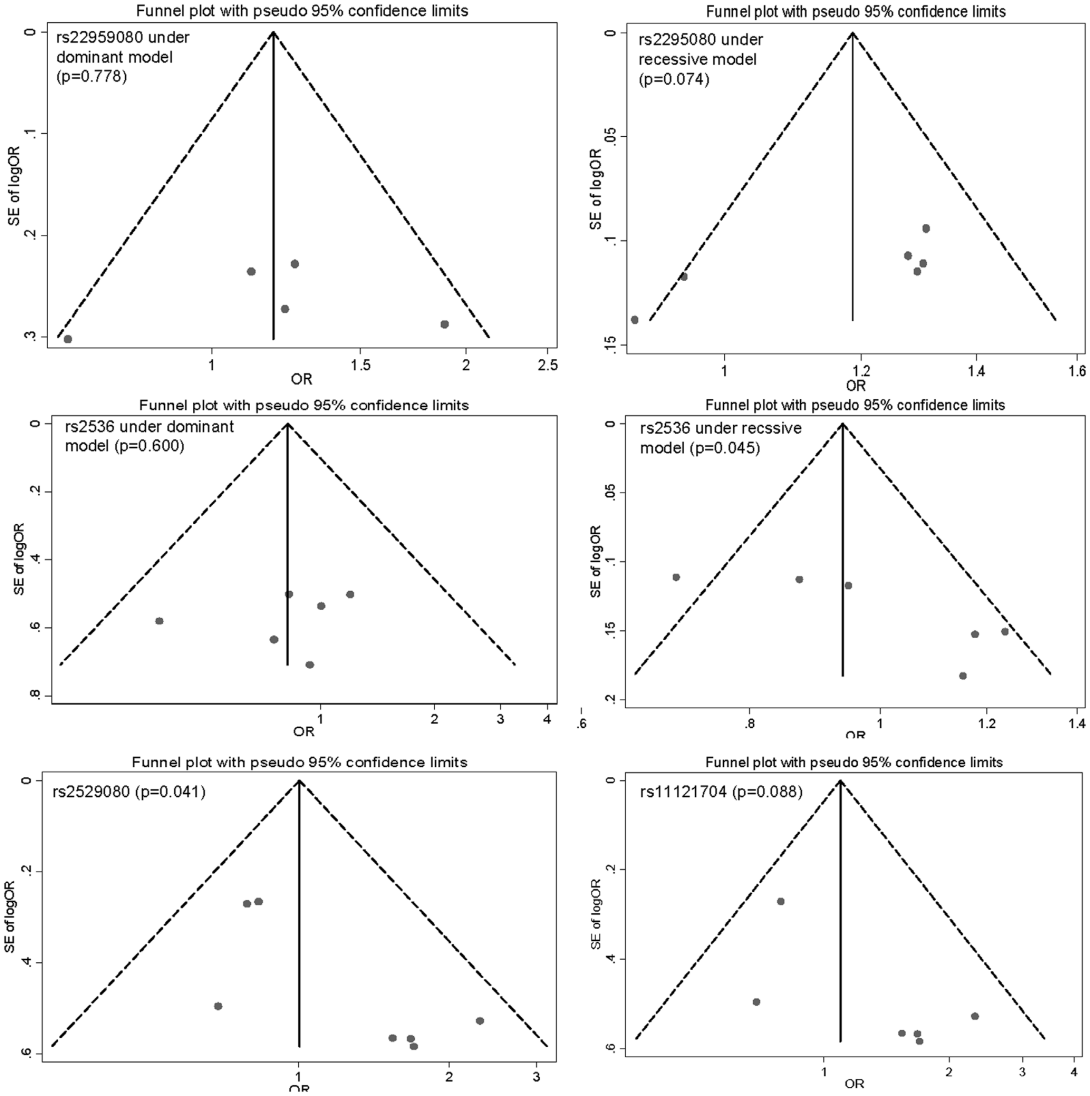
Funnel plots to detect publication bias. Each point represents an independent study for the indicated association.

Begg's funnel plot and Egger's test were performed to evaluate the publication bias of literatures. The shapes of the funnel plots did not reveal any evidence of obvious asymmetry except for the association of rs2295080 and rs2536 with cancer risk under the recessive model, and the Egger's test also suggested that there was slight publication bias for the latter (p = 0.045) (Fig. 4). In addition, although symmetrical funnel plots were obtained under the recessive model for the association of mTOR polymorphisms with clinical outcomes, the Egger's test indicated publication bias was present for rs2295080 polymorphism (p = 0.041) (Fig. 4). After adjusted by “trim and fill” method did not significantly influence the results from our meta-analysis (OR = 0.99, 95%CI: 0.52–1.47).

## Discussion

This meta-analysis examined the association between the common genetic polymorphisms and cancer risks as well as clinical outcomes. A total of 5798 cancer patients and 6244 controls were included for cancer risk assessment and 1928 cancer patients were included for clinical outcome assessment. We found that the wild genotype TT of rs2295080 polymorphism were associated with increased cancer risk and rs11121704 TT genotype was associated with poor clinical outcomes, such as death, metastasis, resistant to chemotherapy, and toxicity. No significant association was found between rs2536 and cancer risk.

Since one group studied for the first time the germline genetic polymorphisms in the PI3K-AKT-mTOR and cancer risk as well as clinical outcomes [19], [32], a number of studies have been performed to explore the possible influence of the genetic variants in this pathway genes on cancer development, progression, and prognosis. In this meta-analysis, we focused on the common polymorphisms in mTOR gene and evaluated their correlation with cancer risk and clinical outcomed in cancer patients. Constitutive activation of the mTOR signaling had been reported in a few human cancers and higher mTOR expression had been observed in tumor tissues compared to corresponding normal tissues [13], [34]. Recently, the rs2295080 polymorphism in the promoter was demonstrated to decrease the transcriptional activity of mTOR in vitro assay, and be associated with lower mTOR mRNA expression in renal tissues [13]. Given the crucial role of mTOR in multiple cellular functions, such as in cell death and survival, as well as angiogenesis, our findings of an association between the rs2295080 and cancer risk are biologically plausible. In addition, high mTOR expression was associated with a poor prognosis in several human cancers, including renal cell cancer, lung cancer, breast cancer, laryngeal squamous cell carcinoma, neuroendocrine tumors, biliary tract adenocarcinoma, and colorectal cancers [6]–[10]. Our meta-analysis results demonstrated that the TT genotype was associated with poor clinical outcome parameters. Since there was no functional study about mTOR rs11121704 polymorphism, thus we used the SNPexp online tool (http://app3.titan.uio.no/biotools/tool.php?app=snpexp) to evaluate the possible biological influence on mTOR gene expression. We found that the individuals with TT genotype had higher mTOR gene expression levels than those individuals with TC and CC genotypes, although not reaching statistical significance (p = 0.059). However, the rs11121704 polymorphism is located in intron, and it is unlikely that the rs11121704 polymorphism exert its effect by modulating mTOR gene expression, thus, additional explanation for this correlation may be due to linkage disequilibrium with other functional polymorphisms. This hypothesis is needed to be tested in future mechanistic studies.

Some limitations of this meta-analysis should be addressed. For the cancer risk assessment, all these studies included in our meta-analysis were conducted in Chinese population, and 5 studies conducted in USA were excluded due to insufficient genotyping data or not examine rs2295080 or rs2536 polymorphisms [27]–[32]. Thus, our findings on the influence of mTOR polymoprhisms on cancer risk only represent Chinese population. Due to small number of studies included in our meta-analysis, we did not stratify these studies by cancer type. In addition, some control subjects in different studies were from same study group [12], [17]–[18], in which the same controls might be matched to different cases. For clinical outcome assessment, some studies were excluded due to insufficient genotyping data available [26], [27], which could affect the final pooled results. In addition, combined different type paramaters of clinical outcomes, e.g., survival, recurrence, and toxicity, may not be appropriate to assess the influences of genetic polymorphisms. Furthermore, two of them reported more than one clinical outcome parameter and these parameters were separately analyzed as separate observations [19]–[20], which could produce publication bias. In spite of these limitations, our meta-analysis also had some advantages. First, quality control for genotyping assay was performed in all studies except for one [20]. Second, the information from these eligible studies is assessed under both dominant and recessive models.

In conclusion,this meta-analysis showed that the mTOR polymorphisms (rs2295080 and rs11121704) were associated with cancer risk and clinical outcomes of cancer patients, respectively, and no any association was found for the rs2536 polymorphism. As all studies included in our meta-analysis for the assessment on cancer risk are limited in Chinese population, even for the evaluation on the clinical outcomes, only four studies conducted in China and USA were included, thus, further studies including a wider spectrum of subjects should be conducted in Caucasians and other ethnicities, which could result in comprehensive understanding of mTOR polymorphisms on cancer risk and the clinical outcomes.

## Supporting Information

Checklist S1 PRISMA checklist.
(DOC)
